# Advanced age and increased CRP concentration are independent risk factors associated with *Clostridioides difficile* infection mortality

**DOI:** 10.1038/s41598-020-71466-0

**Published:** 2020-09-07

**Authors:** Agnieszka Bednarska, Dominik Bursa, Regina Podlasin, Marcin Paciorek, Agata Skrzat-Klapaczyńska, Dawid Porowski, Joanna Raczyńska, Joanna Puła, Dominika Krogulec, Michał Makowiecki, Andrzej Horban

**Affiliations:** 1 Department of Adults’ Infectious Diseases, Medical University of Warsaw, Żwirki i Wigury 61, 02-091 Warsaw, Poland; 2 Hospital for Infectious Diseases, Wolska 37, 01-301 Warsaw, Poland

**Keywords:** Bacteriology, Clostridium difficile

## Abstract

*Clostridioides difficile* (*C*.*difficile*) is a Gram-positive, spore-forming, toxin-producing anaerobic bacillus, which is one of the most common causes of health-care-associated infection developed mainly by elderly patients. The objective of this study was to assess mortality among the patients of the Hospital for Infectious Diseases in Warsaw related to *C.difficile* infection. Analysis was conducted of 1638 records reporting the medical histories of patients hospitalized for the first time due to *Clostridioides difficile infection* (CDI) in the Hospital for Infectious Diseases in Warsaw from 2010 to 2017. The inclusion criteria were any (principal or secondary) discharge diagnosis code for CDI according to ICD-10 and being an adult (≥ 18 years). 108 out of 1638 (7%) of the patients died. The median age in this group was 83 years. The largest number of deaths (90%) occurred in the group of patients aged 65 years or older and 81–90 years old (53% of all the deaths). In the multivariate logistic regression model relevant only to the age groups, not to sepsis—age over 80 and over 90 were independent predictors of death, increasing the risk of death by 3.4 and 1.8 times, respectively. The result of the receiver operating curve (ROC) analysis determined the age of 77 years as the threshold value, indicating the increased risk of death (AUC 0.727, standard error 0.025, 95% CI 0.678–0.776, *p* < 0.0001). In addition, other quantitative variables, namely CRP, creatinine and leucocytes were studied and turned out to be independent death predictors as well. The diagnosis of sepsis increased the risk of death fourfold (OR = 4.042; 95% Cl 2.4–6.7; *p* < 0.001). Increased inflammatory parameters, namely CRP and white blood cell count, advanced age, particularly over the age of 80, as well as a diagnosis of sepsis are independent risk factors for death and could be used as predictive markers of poor outcome in CDI.

## Introduction

*Clostridioides difficile* (*C.difficille*) is a Gram-positive, spore-forming, toxin-producing anaerobic bacillus, which is one of the most common causes of health-care-associated infection developed mainly by elderly patients^1–3^. *Clostridioides difficile* infection (CDI) is characterized by a wide spectrum of clinical symptoms from mild diarrhea to fulminant and fatal toxic colitis and is associated with a 50% increased risk of death^4–6^. The high level of inflammatory parameters is a well-known risk factor for severe CDI and in consequence—mortality^2,7,8^. Old age correlates with severity and the poor outcome of the infection. According to numerous studies, patients aged 65–75 years are at a particularly high risk of death^7–9^.

The objective of this study was to assess mortality among the patients of the Hospital for Infectious Diseases in Warsaw related to *C. difficile* infection and the risk of death in the whole group examined. In addition, analysis was conducted of a breakdown by age among adult and elderly adult patients.

## Methods

We reviewed the records of 1638 patients with CDI who were hospitalized for the first time in the Hospital for Infectious Diseases in Warsaw from 2010 to 2017. Patients were included if they had any (principal or secondary) discharge diagnosis code for CDI according to the International Statistical Classification of Diseases and Related Health Problems (ICD-10) and were adult (≥ 18 years).

The principal diagnosis indicated the main reason for hospitalization according to the assessment of the discharging clinician. CDI was the secondary diagnosis if another disease entity was acknowledged as the basic reason for hospitalization or in case complications occurred in the course of CDI (such as sepsis or death).

The procedure adopted in the Hospital for Infectious Diseases in Warsaw made it possible to diagnose *C. difficile* infection in patients with diarrhea and positive result of *C. difficile* antigen or/and toxin A/B enzyme immunoassay or nucleic acid amplification test. Due to the change of the definition of sepsis, until the end of 2016 the ICD-10 code for sepsis was assigned to persons with diagnosed CDI and the presence of systemic inflammatory response and since 2017 the presence of organ disfunction was recorded.

They were categorized into age groups as follows: adults (18–64 years), elderly adults (≥ 65 years), persons aged 18–20 years, 21–30 years, then with intervals of every 10 years (i.e. 31–40, 41–50, etc.). The last group comprised patients over 100 years old. The number of deaths, both in the whole group of patients and in particular subgroups, were analyzed. Additionally, the Optimed database Esa Project, Poland) was searched for initial (admission) results of leukocyte count, CRP and creatinine level.

### Statistical calculations

The distribution of variables was analyzed with the Shapiro–Wilk test. In the absence of a normal distribution of variables, the results were presented as median values and interquartile ranges, (Md, IQR). The Mann–Whitney U test was used to compare the groups. Variables associated with an increased probability of death were identified in the logistic regression analysis. Multivariate logistic regression analysis was used to determine independent predictors of death. Construction of the multivariate model was based only on those variables whose *p* level of significance in univariate analysis was not greater than 0.2. Evaluation of all the effects was used for building the model. A receiver operating characteristic curve (ROC), area under the curve (AUC) and Youden’s index were applied to identify the optimal cut-off point of the variables’ sensitivity and specificity. The analyses were based on the non-parametric method according to Hanley and McNeil^10^. The level of statistical significance was *p* < 0.05. All the analyses were performed using the Statistica software, version 13.1, StatSoft with a medical add-on.

### Consent for publication

Consent of the Komisja Bioetyczna (Bioethical Commission), Medical University of Warsaw no AKBE 112/2019. Due to the retrospective nature of the study, the bioethics committee did not require the researchers should obtain informed consent from the subjects whose data were analyzed.

## Results

Over the study period, 1638 (1,016 women and 622 men) patients were included for analysis. The median age of the researched population was 75 (22), the minimum age was 18, while the maximum—101 years. Of these persons 484 (30%) were adults and 1,154 (70%) were elderly adults. Considering the 10-year interval division, persons aged 71–90 were most commonly hospitalized (55% of all the patients studied) (Table 1).

**Table 1:** Demographic data of the patients included in the study due to their discharge diagnosis of *Clostridioides difficile* infection (CDI).

Age	Total				18–64 years				65–101 years		
No of patients (%)	1,638				484 (30)				1,154 (70)		
Female/male ratio (%)	1,016/622 (62/38)				302/182 (62/38)				714/440 (62/38)		
No of deaths (%)	108 (7)				11 (10% of total no of death)				97 (90% of total no of death)		
Female/male ratio (%)	75/33 (69/31)				8/3 (73/27)				67/30 (69/31)		
Number of patients and deaths in 10-year intervals											
	18–20	21–30	31–40	41–50		51–60	61–70	71–80	81–90	91–100	> 100
No of patients	8	72	99	66		152	271	431	473	64	2
No of deaths (% of total no of deaths)	0 (0)	1 (1)	2 (2)	2 (2)		4 (4)	7 (6)	21 (19)	57 (53)	14 (13)	0 (0)

Seven percent (108 out of the 1638 patients) died. The largest number of deaths (90%) occurred in the group of patients aged 65 years or older and in the group aged 81–90 years (53% of all deaths).

Altogether 1638 patients were included for analysis. The minimum age was 18, and the maximum—101 years. Of these persons 484 (30%) were adults and 1,154 (70%) were elderly adults.

Sepsis was diagnosed in 119 out of the 1638 persons studied (in 106 patients till the end of 2016 according to the older definition and in 13 since the beginning of 2017, according to the latest one).

Seven percent, namely 108 out of the 1638 patients, died. The median age in this group amounted to 83 (9.5%), the minimum age was 29 and the maximum was 94 years. The largest number of deaths (90%) occurred in the group of patients aged 65 years or older and in the group aged 81–90 years (53% of all deaths) (Table 1).

Older age was an independent, significant death risk factor (*p* < 0.001).

Moreover, 23 out of the 108 patients who died were diagnosed with sepsis according to the ICD-10 classification. The largest number of patients in this group were people aged 81–90 years (11 patients, 48%).

In univariate and multivariate logistic regression analysis, the diagnosis of sepsis increased the risk of death fourfold (OR = 4.042; 95% Cl 2.4–6.7; *p* < 0.001 and OR = 4.04; 95% Cl 2.4–6.9; *p* < 0.001 respectively).(Table 2).

**Table 2:** Univariate and multivariate logistic regression analysis of sepsis and age as death risk factors among the 1638 patients included in the study due to their discharge diagnosis of *Clostridioides difficile* infection (CDI).

	OR	95% CI	*p*
**Association with mortality in univariate logistic regression analysis**			
ICD-10 code for sepsis in diagnosis at discharge	4.042	2.440–6.697	< 0.001
Age ≥ 70 years	4.19	2.406–7.297	< 0.001
Age ≥ 80 years	4.93	3.197–7.616	< 0.001
Age ≥ 90 years	3.87	2.246–6.667	< 0.001
**Association with mortality in multivariate logistic regression analysis**			
ICD-10 code for sepsis in diagnosis on discharge	4.04	2.373–6.875	< 0.001
Age ≥ 70 years	1.68	0.807–3.486	0.166
Age ≥ 80 years	3.34	1.858–6.016	< 0.001
Age ≥ 90 years	1.68	0.938–3.011	< 0.001

*ICD-10* International Statistical Classification of Diseases and Related Health Problems, *OR* odds ratio, *CI* confidence interval, *p* probability value.

Sepsis was diagnosed in 119 out of 1638 persons (in 106 patients till the end of 2016 according to the older definition and in 13 since the beginning of 2017, according to the latest one). In the whole studied group, 108 persons died, 23 of them ICD -10 code for sepsis was assigned. In univariate and multivariate logistic regression analysis, the diagnosis of sepsis increased the risk of death fourfold.

The model of multivariate analysis consisting of a sepsis diagnosis and age revealed that sepsis, age over 80 years and (to a lesser degree) age over 90 years were independent death risk factors (Table 3).

**Table 3:** Univariate and multivariate logistic regression analysis of age as a risk factor with the categorization of the 1638 studied patients into age subgroups with intervals of every 10 years.

	OR	95% CI	P
**Association with mortality in univariate logistic regression analysis**			
Age ≥ 40 years	4.26	1.337–13.573	0.014
Age ≥ 50 years	3.74	1.507–9.271	0.004
Age ≥ 60 years	3.48	1.743–6.962	< 0.001
Age ≥ 70 years	4.19	2.406–7.297	< 0.001
Age ≥ 80 years	4.93	3.197–7.616	< 0.001
Age ≥ 90 years	3.87	2.246–6.667	< 0.001
**Association with mortality in multivariate logistic regression analysis**			
Age ≥ 40 years	1.604	0.262–9.811	0.609
Age ≥ 50 years	1.038	0.185–5.807	0.966
Age ≥ 60 years	0.801	0.222–2.889	0.735
Age ≥ 70 years	1.617	0.619–4.223	0.327
Age ≥ 80 years	3.380	1.887- 6.055	< 0.001
Age ≥ 90 years	1.803	1.021- 3.186	0.042

*ICD-10* International Statistical Classification of Diseases and Related Health Problems, *OR* odds ratio, *CI* confidence interval, *p* probability value.

In this analysis only age, not the sepsis diagnosis was considered. Age over 80 years and (to a lesser degree) age over 90 years were independent death risk factors.

In the multivariate logistic regression model, relevant only to the age groups, not to sepsis—age over 80 and over 90 were independent predictors of death, increasing the risk of death by 3.4 and 1.8 times, respectively (Table 3).

In addition, the other quantitative variables that were analyzed, namely CRP, creatinine and leucocytes turned out to be independent death predictors as well (Table 4).

**Table 4:** Univariate and multivariate logistic regression analysis of the 1638 patients included in the study due to their discharge diagnosis of *Clostridioides difficile* infection with age and initial (admission) results of inflammatory parameters as death risk factors.

	OR	95% CI	*p*
**Association with mortality in univariate logistic regression analysis**			
Age	1.061	1.041–1.082	< 0.001
CRP	1.006	1.005–1.008	< 0.001
creatinine	1.007	1.005–1.009	< 0.001
Leukocytes	1.057	1.040–1.074	< 0.001
**Association with mortality in multivariate logistic regression analysis**			
Age	1.053	1.032- 1.075	< 0.001
CRP	1.005	1.003–1.007	< 0.001
creatinine	1.004	1.001–1.006	< 0.001
Leukocytes	1.029	1.014–1.044	< 0.001

CRP C-reactive protein, OR odds ratio, CI confidence interval, *p* probability value.

All the quantitative variables that were analyzed turned out to be independent death predictors.

The median length of hospitalization (LOS) in the group of the deceased patients was 10 (13) days (min–max: 1–180), while in the group of survivors 11 (9) days (min–max: 1–207), *p* = 0.02. Sex had no impact on the risk of death (*p* = 0.1018) in the univariate analysis.

**Figure 1:**
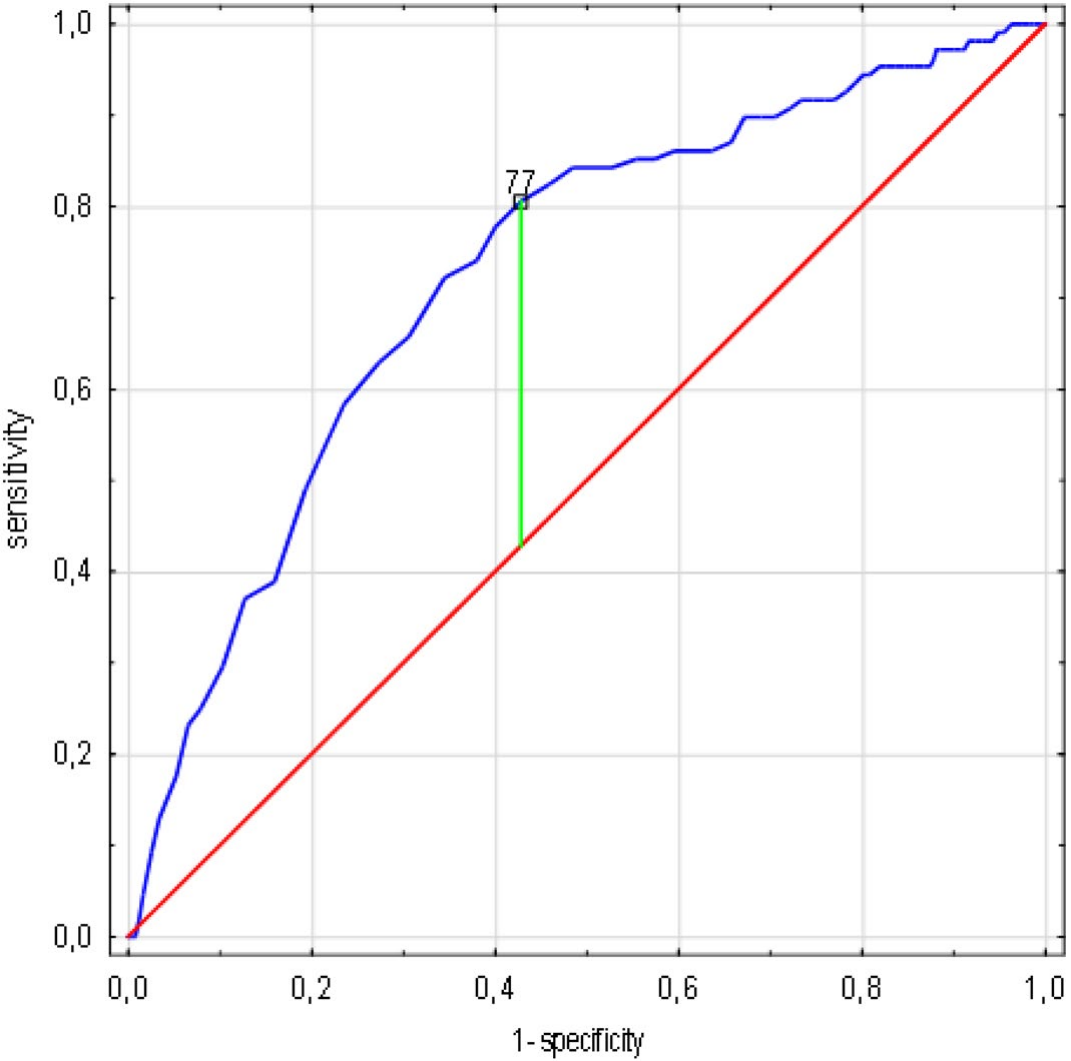
Age cut-off value associated with increasing risk of death in ROC analysis. (n = 1638 patients/108 died). Legend: sensitivity (True Positive Rate), 1-specificity (False Positive Rate); scale is in decimals of sensitivity and specificity; interval every 0.2, i.e. 20%; green line—optimum cut-off point (max. Youden’s index = 0,38, cut-off point: 77,00), blue line—ROC curve with AUC 0.727, 95%CI 0.678–0.776, SE 0.025, *p* < 0.001, red line—classification due to chance with AUC 0.5. Abbreviations: ROC—receiver operating characteristic, AUC—area under the curve, SE—standard error. Method: ROC, AUC and SE analyzes performed according to Hanley and McNeil^10^.

The result of the receiver operating curve (ROC) analysis determined the age of 77 years as the threshold value, indicating an increased risk of death (AUC 0.727, standard error 0.025, 95% CI 0.678–0.776, *p* < 0.0001) (Fig. 1).

**Figure 2:**
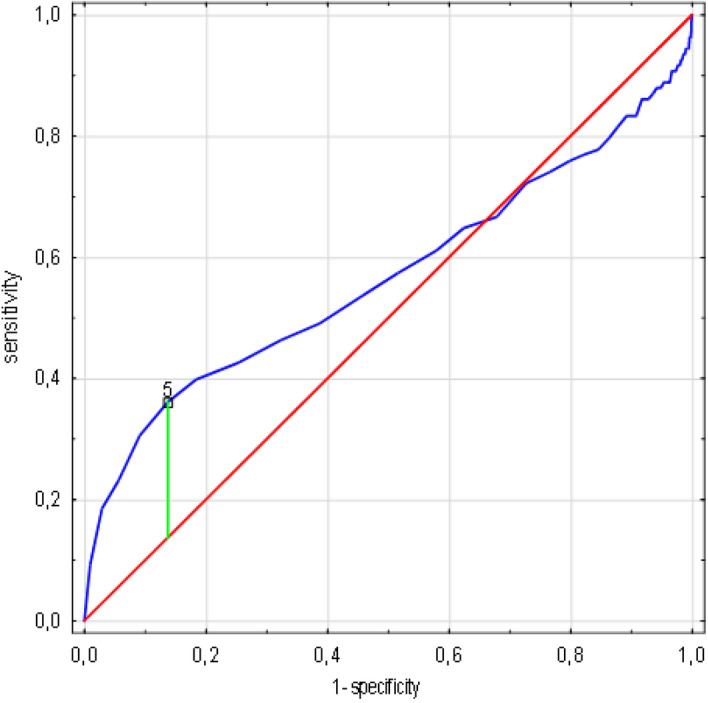
LOS cut-off value associated with increasing risk of death in ROC analysis. (n = 1638 patients/108 died). Legend: sensitivity (True Positive Rate), 1-specificity (False Positive Rate); scale is in decimals of sensitivity and specificity; interval every 0.2, i.e. 20%; green line—optimum cut-off point (max. Youden’s index = 0.22, cut-off point: 5), blue line—ROC curve with AUC 0.567, 95%CI 0.498–0.637, SE 0.035, *p* = 0.0568, red line—classification due to chance with AUC 0.5. Abbreviations: ROC—receiver operating characteristic, AUC—area under the curve, SE—standard error, LOS—length of stay. Method: AUC, ROC and SE analyzes performed according to Hanley and McNeil^10^.

The risk of death was calculated as increasing by one percent per each day of hospitalization (OR 1.012, 95% CI 0.999–1.024, *p* = 0.0642). The fifth day was the most sensitive and specific cut-off point in the ROC analysis (AUC 0.567, SE 0.035, 95% CI 0.498–0.637, *p* = 0.0568), however none of these results was statistically significant (Fig. 2).

**Figure 3:**
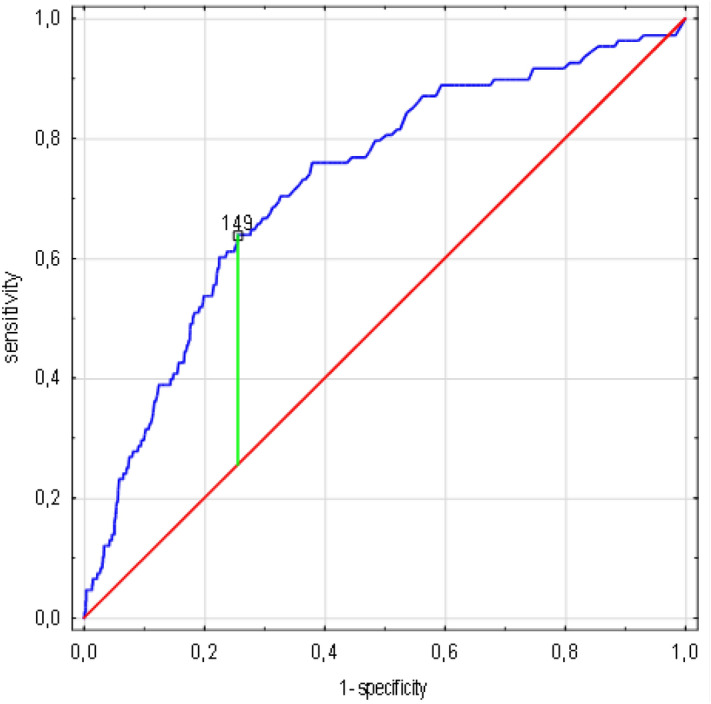
CRP concentration cut-off value associated with increasing risk of death in ROC analysis. (n = 1638 patients/108 died). Legend: sensitivity (True Positive Rate), 1-specificity (False Positive Rate); scale is in decimals of sensitivity and specificity; interval every 0.2, i.e. 20%; green line—optimum cut-off point (max. Youden’s index = 0.38, cut-off point: 149), blue line—ROC curve with AUC 0.725, 95%CI 0.674–0.776, SE 0.026, *p* < 0.001, red line—classification due to chance with AUC 0.5. Abbreviations: ROC—receiver operating characteristic, AUC—area under the curve, SE—standard error, CRP—C-reactive protein. Method: AUC, ROC and SE analyzes performed according to Hanley and McNeil^10^.

**Figure 4:**
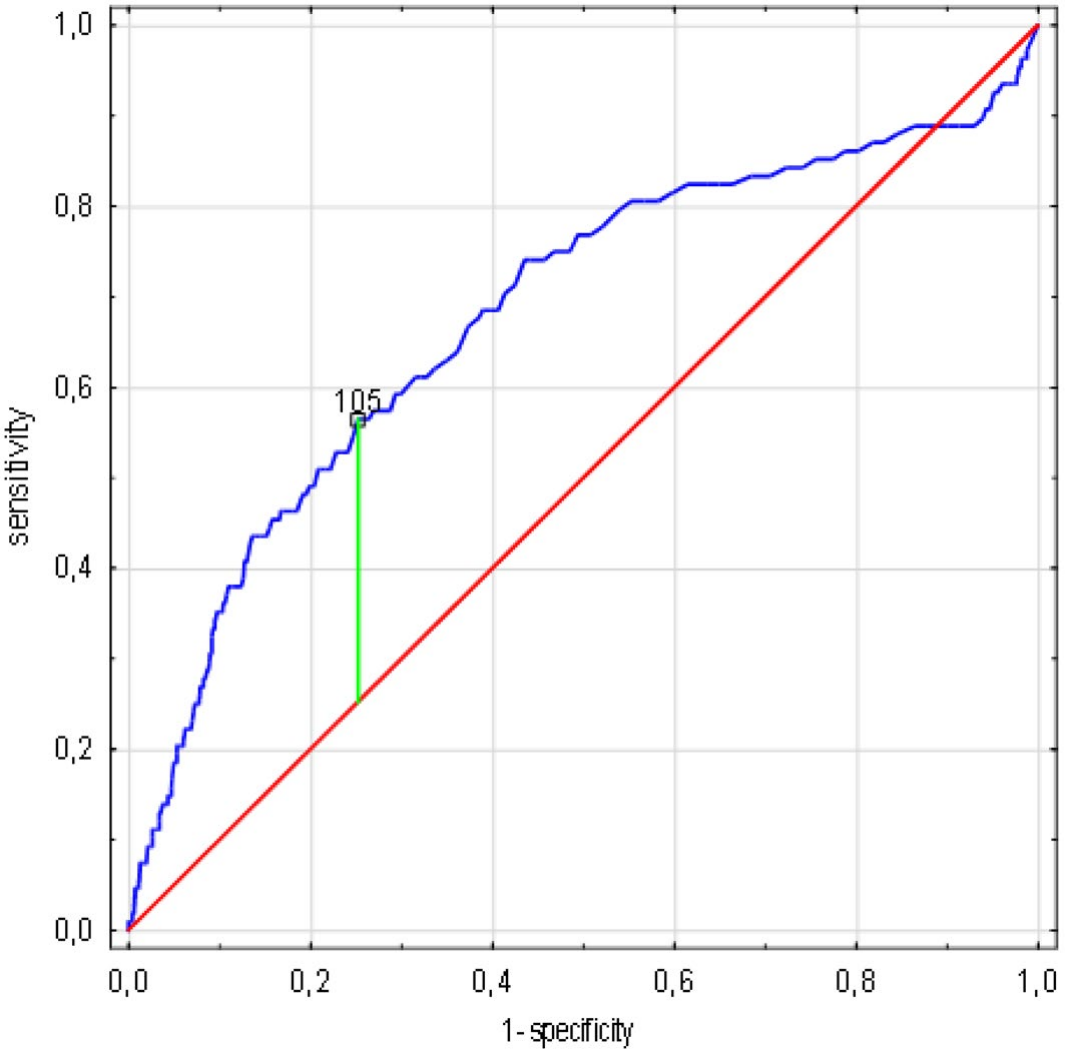
Creatinine concentration cut-off value associated with increasing risk of death in ROC analysis. (n = 1638 patients/108 died). Legend: sensitivity (True Positive Rate), 1-specificity (False Positive Rate); scale is in decimals of sensitivity and specificity; interval every 0.2, i.e. 20%; green line—optimum cut-off point (max. Youden’s index = 0,31, cut-off point: 105), blue line—ROC curve with AUC 0.68, 95%CI 0.613 -0.741, SE 0.03, *p* < 0.001, red line—classification due to chance with AUC 0.5. Abbreviations: ROC—receiver operating characteristic, AUC—area under the curve, SE—standard error. Method: AUC, ROC and SE analyzes performed according to Hanley and McNeil^10^.

**Figure 5:**
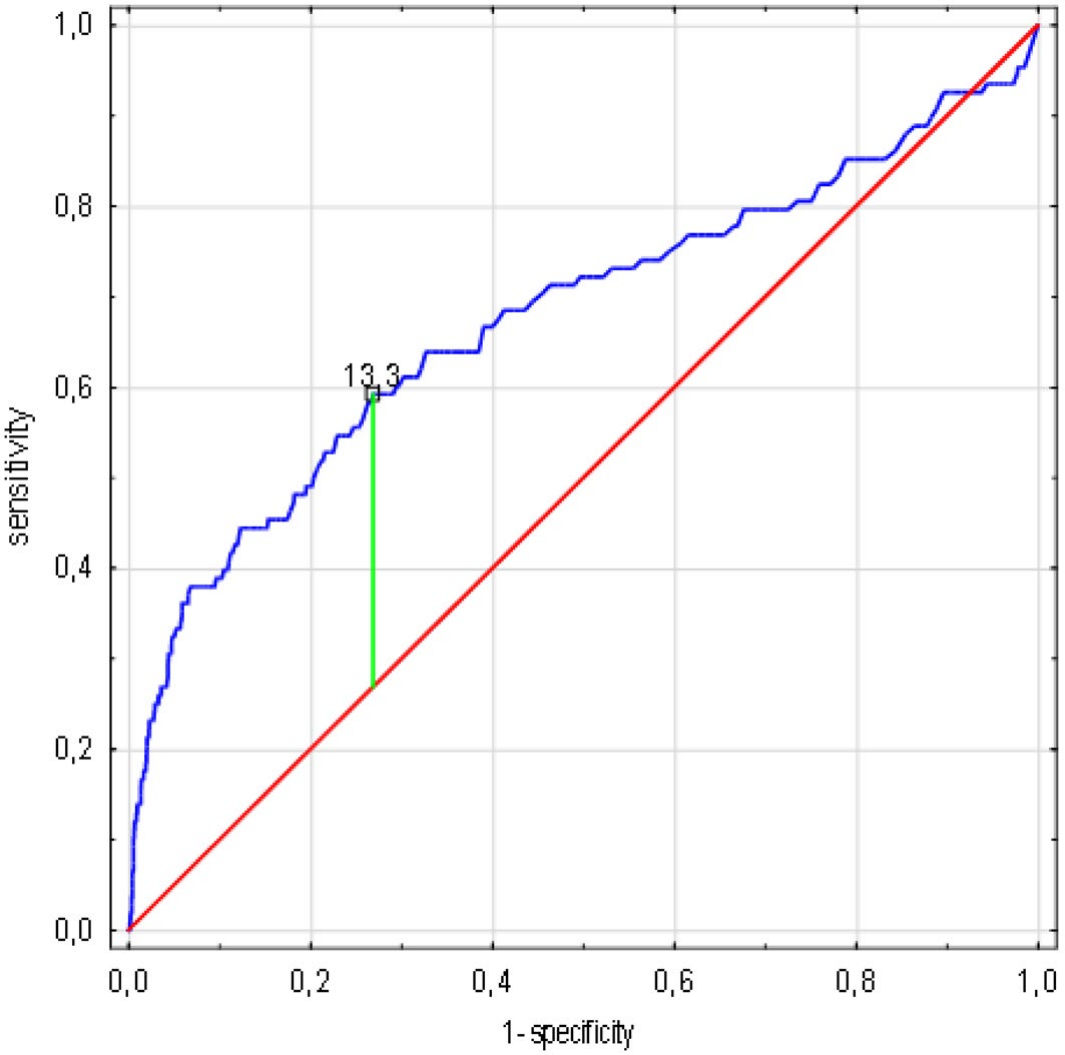
Leucocyte count cut-off value associated with increasing risk of death in ROC analysis. (n = 1638 patients / 108 died). Legend: sensitivity (True Positive Rate), 1-specificity (False Positive Rate); scale is in decimals of sensitivity and specificity; interval every 0.2, i.e. 20%; green line—optimum cut-off point (max. Youden’s index = 0,32, cut-off point: 13,3), blue line—ROC curve with AUC 0.677, 95%CI 0.62 -0.739, SE 0.032, *p* < 0.001, red line—classification due to chance with AUC 0.5. Abbreviations: ROC-receiver operating characteristic, AUC-area under the curve, SE-standard error. Method: AUC, ROC and SE analyzes performed according to Hanley and McNeil^10^.

The result of the receiver operating curve (ROC) analyses showed that in the case of CRP concentration, the cut-off value associated with increasing the risk of death was 149 mg/l (AUC 0.725, standard error 0.026, 95% CI 0.674 -0.776, *p* < 0.0001), for the creatinine level 105 µmol/L (AUC 0.68, SE 0.03, 95% CI 0.613 -0.741, *p* < 0.0001) and for the leukocyte count 13 300/µL (AUC 0.677, SE 0.032, 95% CI 0.62 -0.739, *p* < 0.0001) (Figs. 3, 4, 5).

## Discussion

The study evaluated 1638 patients. One hundred and eight of them died. Age, sex, length of hospitalization and basic laboratory test results were analyzed in the dead patients’ group and in the group of survivors. The findings indicated that age and the level of CRP, creatinine and leukocytes were statistically higher in the group of the deceased patients. The results were confirmed with multivariate logistic regression analyses. Age and the laboratory parameters analyzed proved to be independent risk factors for death.

There are many studies demonstrating old age as a risk factor for complicated *C. difficile* infection outcomes^8,11–13^. Patel et al.^14^ narrowed the age range to 70 or more years as severe CDI-risk-related and Khanna et al.^4^ showed that patients suffering from severely complicated infection were significantly older, with the median age of 80 years. In the study by Morrison RH et al., the analyses were limited to mortality alone and indicated independently significant associations between age ≥ 80 years and death, with an odds ratio of 5.5^15^.

In our study, the significant risk of death occurred among patients older than 80 years. Patients aged 91–100 years were burdened with a more than five times greater risk compared with the rest of the groups studied. The age of 77 years appeared to be the best cut-off value, indicating the increased risk of death in ROC analyses.

In this research, we also evaluated the impact of basic laboratory parameter results on mortality. CRP and creatinine concentration, as well as leukocyte count, were higher in the group of patients who died versus the group of survivors (*p* < 0.001) and together with age were independent risk factors for death. All the laboratory results obtained on admission were evaluated and submitted for statistical analysis, including receiver operating curve analyses. No division into groups depending on the result and no cut-off point were introduced. This can be the reason for lower median values, lower ORs and a comparatively low cut-off value in ROC analysis compared to other studies^2,8,16^.

C-reactive protein is an acute phase response protein, secreted by hepatocytes under the influence of proinflammatory cytokines, especially interleukin-6^17,18^. Its level increases during bacterial infection^19^. The activation of the auto-amplifying cytokine production process, the cytokine storm, determines the severity of infection and eventually leads to sepsis. Among the pro-inflammatory cytokines studied during sepsis, IL-1β, IL-6, IL-12, and IL-17 are of crucial importance^20^. In our study, patients with an assigned ICD-10 sepsis code had a fourfold higher odds probability of death. Although CRP concentration is neither considered a parameter indicating severe CDI, nor is it included in the sequential organ failure assessment (SOFA) score^7,21^, it was added to our analysis, giving the result of increasing the risk of death by 50% with every 100 mg/L accretion of CRP.

The study has several limitations, such as its retrospective character, or the lack of determining the time from diagnosis to death, but it also features advantages in the form of a clearly selected, numerous group of patients, with mortality assessed only during the first hospitalization in the Hospital for Infectious Diseases due to CDI.

Therefore, we can conclude that advanced age, particularly over 80 years, increase in inflammatory parameters, namely CRP and white blood cells as well as a diagnosis of sepsis are independent risk factors for death and could be used as predictive markers of CDI poor outcome.
